# Integrated III-V Photonic Crystal – Si waveguide platform with tailored optomechanical coupling

**DOI:** 10.1038/srep16526

**Published:** 2015-11-16

**Authors:** Viktor Tsvirkun, Alessandro Surrente, Fabrice Raineri, Grégoire Beaudoin, Rama Raj, Isabelle Sagnes, Isabelle Robert-Philip, Rémy Braive

**Affiliations:** 1 Laboratoire de Photonique et Nanostructures LPN-CNRS UPR-20, Route de Nozay, 91460 Marcoussis, France; 2 Université Paris Diderot, F-75205 Paris Cedex 13, France

## Abstract

Optomechanical systems, in which the vibrations of a mechanical resonator are coupled to an electromagnetic radiation, have permitted the investigation of a wealth of novel physical effects. To fully exploit these phenomena in realistic circuits and to achieve different functionalities on a single chip, the integration of optomechanical resonators is mandatory. Here, we propose a novel approach to heterogeneously integrate arrays of two-dimensional photonic crystal defect cavities on top of silicon-on-insulator waveguides. The optomechanical response of these devices is investigated and evidences an optomechanical coupling involving both dispersive and dissipative mechanisms. By controlling the optical coupling between the waveguide and the photonic crystal, we were able to vary and understand the relative strength of these couplings. This scalable platform allows for an unprecedented control on the optomechanical coupling mechanisms, with a potential benefit in cooling experiments, and for the development of multi-element optomechanical circuits in the framework of optomechanically-driven signal-processing applications.

Cavity optomechanics explores the coupling between the degrees of freedom of a mechanical oscillator and those of an optical or a microwave mode, allowing for optical sensing and control of the mechanical vibrations and vice-versa^1^,^2^,^3^. Explored in a wide variety of systems ranging from Fabry-Pérot interferometers^4^ to Fabry-Pérot cavities with moving micromirrors or embedding vibrating membranes^5^,^6^,^7^,^8^,^9^,^10^, this field has extended towards nanoscale implementations with chip-scale optomechanical devices mostly inspired from largely explored nanophotonic platforms. Such nanoscale devices include microdisks^11^,^12^, on-chip waveguides^13^,^14^, plasmonic cavities^15^, as well as photonic and phoxonic crystal resonators^16^,^17^,^18^,^19^,^20^,^21^. The applications of these optomechanically driven platforms are strongly enhanced by the integrability of nanoscale optomechanical systems on a single chip setting, paving the way to multielement optomechanical platforms. Beyond opening new avenues in experimental quantum mechanics or in quantum information processing^22^,^23^,^24^,^25^,^26^,^27^, multimode optomechanics holds promise for enhanced performance in metrology^28^,^29^,^30^,^31^ or for new on-chip functionalities for signal processing, such as information storage^32^,^33^, and wavelength conversion^34^,^35^.

The implementation of such multichannel and multimode circuits requires an efficient light coupling at the nanoscale and the combination on a single chip of interacting optomechanical elements with a high design flexibility. Most current experiments in this direction rely on the planar integration of the various functionalities ensuring light coupling, signal detection, amplification, storing or processing^36^,^37^,^38^,^39^,^40^. In this article, a complete three-dimensional (3D) integration is achieved by relying on a combined bottom-up and top-down approach, as opposed to most schemes, solely based on a top-down processing. This hybrid 3D integration is demonstrated by vertically stacking an array of standalone InP-based optomechanical resonators on top of low-loss silicon-on-insulator (SOI) optical waveguides [see Fig. 1(a) for a schematic illustration of a single device]. This highly flexible approach allows in principle for the heterogeneous integration of a wide variety of materials, with specific combinations depending on the targeted applications. Additional functionalities might also be integrated and efficiently coupled to the optomechanical resonators such as electrostatic^41^,^42^ or surface acoustic wave^43^ transducers for resonant mechanical excitation. We demonstrate that these hybrid fully-integrated optomechanical PhC cavities form systems in which the position of the mechanical oscillator modulates both the resonant frequency (dispersive coupling) and the linewidth (dissipative coupling) of the resonator. A fine tailoring of both optomechanical coupling mechanisms through their dependence on the 3D platform geometry (waveguide width and waveguide-PhC cavity distance) is also shown. Such fine tailoring represents a first, required step towards novel experiments on the optical cooling of the mechanical vibrations in the unresolved-sideband regime^44^. The integrated nature of the implemented optomechanical platform also paves the way to highly-flexible, tunable 3D optomechanical circuits with arbitrary configurations enabling various and more complex on-chip architectures.

## Results

### Basic optomechanical characterization

**Figure 1:**
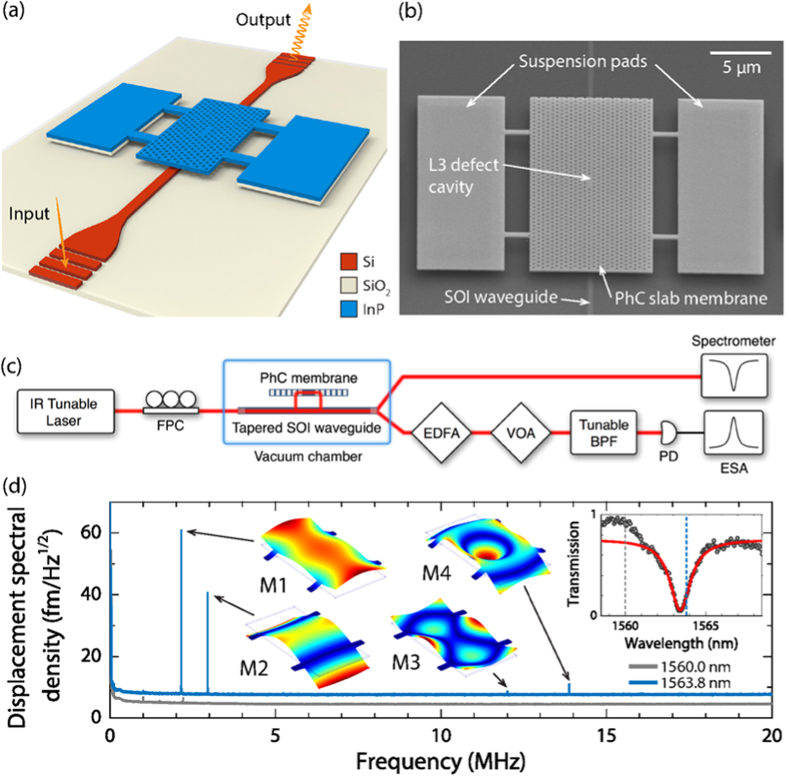
Optomechanics with heterogeneously integrated InP PhC cavities. (**a**) Schematic illustration of an integrated PhC mechanical resonator vertically stacked over a SOI waveguide. The mechanical resonator is the suspended PhC membrane, whose motion can be detected over a broad bandwidth by measuring the optical transmission of the waveguide. (**b**) Scanning Electron Microscope image of a fabricated device. (**c**) Schematics of the experimental setup used for the detection of the mechanical modes. FPC: fiber polarization controller, PhC: photonic crystal, EDFA: erbium-doped fiber amplifier, VOA: variable optical attenuator, BPF: band-pass filter, PD: photodiode, ESA: electrical spectrum analyzer. (**d**) Wide range, calibrated displacement spectra 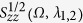 of a PhC mechanical resonator, measured on (blue curve) and off (grey curve) the PhC cavity resonance, respectively. The mechanical mode patterns as obtained from finite element modelling are depicted. Inset: transmission spectrum of the PhC cavity mode (grey circles) and Lorentzian fit (solid red line). The wavelengths used for the measurements of the displacement spectra are indicated by dashed vertical lines.

The investigated sample consists of two-dimensional photonic crystal (PhC) defect cavities etched into a thin InP membrane, heterogeneously integrated onto a SOI waveguide substrate (see Methods for additional details on the fabrication process). The optical resonator consists of a modified L_3_ PhC cavity^45^ (see Methods for details on the PhC cavity design). The mechanical resonator is formed by the suspended PhC slab (lateral size 10 μm × 20 μm, measured suspension height *h* = 230 nm, allowing for evanescent coupling of the guided light into the PhC cavity), connected to the two lateral InP suspension pads through four (1 μm wide, 2 μm long) bridges, as shown in Fig. 1(b). The fabricated devices are first characterized optically [see Fig. 1(c) for a schematic of the experimental setup and Methods for a detailed description]. In the inset of Fig. 1(d), a typical, normalised transmission spectrum *T*(*λ*) is displayed. This PhC cavity presents a resonance at 1563.42 nm, with an optical loaded *Q*_o_ ~ 1100, limited by the losses introduced by the evanescent coupling of the PhC cavity mode to the waveguide mode. When the laser is tuned in the proximity of the PhC cavity resonance, the Brownian motion of the InP membrane results in an intensity modulation of the laser light signal output from the waveguide. The vibration mode spectrum of the membrane can be accessed by measuring the radiofrequency spectral noise of the transmitted light. The wide range mechanical spectrum of an InP membrane resonator is obtained by setting the laser wavelength to *λ*_1_ = 1563.8 nm, slightly detuned to the red side with respect to the PhC cavity resonance [see blue dashed line in the inset of Fig. 1(d)]. The calibrated displacement spectrum 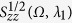
46 of Fig. 1(d) is composed of four narrow peaks (labelled M1 to M4) in the MHz range, exhibiting mechanical quality factors *Q*_m_ ~ 2000–3000. Such modes correspond to flexural modes, whose displacement patterns involve the motion of the full InP membrane. The computed mode displacement patterns shown in Fig. 1(d) are assigned to the respective mode peaks, based on Finite Element Modelling (FEM). To confirm that the peaks observed in 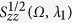 were indeed to be attributed to mechanical modes of the vibrating InP membrane, the probe laser was tuned to *λ*_2_ = 1560 nm, off the PhC cavity resonance by ~3.5 nm [see grey dashed line in the inset of Fig. 1(d)]. The corresponding displacement spectrum 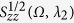 is displayed as the grey curve in Fig. 1(d).

### Optomechanical coupling mechanisms

Optomechanical coupling in our devices arises from four effects. The membrane motion modulates the air gap between the PhC cavity and the waveguide. This affects (i) the resonance wavelength *λ*_c_ of the PhC cavity, which is modified by the presence of the waveguide in the evanescent tail of the PhC cavity, leading to (external) dispersive optomechanical coupling^1^. Such modulation of the air gap also impacts (ii) the optical loss rate of the PhC cavity *κ*_e_ into the access waveguide. This effect, related to a modulation of the overlap between the PhC cavity mode and the waveguide mode, is referred to as (external) dissipative or reactive optomechanical coupling^47^,^48^,^49^,^50^. Finally, the deformation of the InP membrane introduces a modulation of both *λ*_c_ and the intrinsic loss rate *κ*_i_ of the PhC cavity. The former mechanism, known as (iii) intrinsic dispersive coupling, is related to the wavelength shift due to moving dielectric boundaries and to the photo-elastic effect^51^. This effect produces a very small contribution with respect to the external dispersive coupling, as deduced from FEM, and will not be considered henceforth. The latter is referred to as (iv) intrinsic dissipative coupling^49^ and originates from the modulation of *κ*_i_ induced by the membrane deformation. From coupled mode theory, it can be shown that the transmission spectrum *T*(Δ) of a microcavity evanescently coupled to an access waveguide has the form


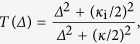


where Δ = *ω* − *ω*_c_ is the detuning between the laser light of frequency *ω* and the cavity mode frequency *ω*_c_, and *κ* = *κ*_i_ + 2*κ*_e_ represents the total cavity loss rate^49^. In the case of the flexural modes considered here, the membrane motion occurs mainly in the out-of-plane (*z*) direction. A slight shift d*z* in this direction induces a variation d*T* in the transmission spectrum given by


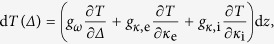


where *g*_*ω*_ = dΔ/d*z* is the external dispersive (dispersive for short in the remainder of the article) optomechanical coupling coefficient, *g*_*κ*,e_ = d*κ*_e_/d*z* is the external dissipative coupling coefficient, and *g*_*κ*,i_ = d*κ*_i_/d*z* is the intrinsic dissipative coupling coefficient^49^. At the mechanical resonance *Ω* = *Ω*_m_, the fluctuations of the transmission spectrum d*T*(Δ)/d*z* contribute directly to the measured power spectral density *S*_P_(*Ω*_m_, Δ) through^46^,^49^


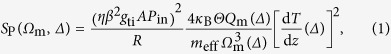


where (for our experimental setup) *η* ~ 0.8 accounts for the coupling efficiency between the laser output and the lens focusing the beam onto the input grating coupler, *β* ~ 0.035 accounts for the coupling efficiency into (and out of) the access waveguide, *A* ~ 25 denotes the signal amplification, *P*_in_ = 6.8 mW designates the probe laser input power, *g*_ti_ = 1400 V/W is the transimpedance gain of the photodetector, *R* = 50 Ω stands for the load resistance, k_B_ is the Boltzmann constant, *Θ* ~ 294 K is the experiment temperature, and *m*_eff_ is the effective mass of the mechanical mode. *Q*_m_(Δ) designates the mechanical mode quality factor, and the mechanical frequency *Ω*_m_(Δ) depends on the probe laser detuning as a result of the optical spring effect^1^.

### Dependence of coupling strengths on waveguide width

**Figure 2:**
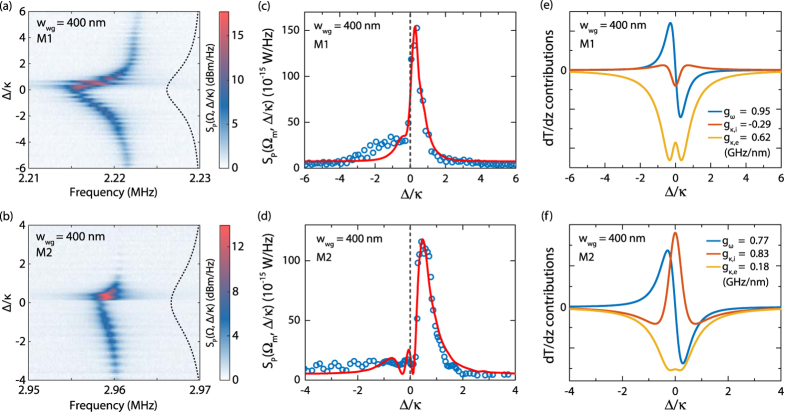
Optomechanical response for a fixed *w*_wg_. Power spectral density *S*_P_(*Ω*, Δ/*κ*) for modes (**a**) M1 and (**b**) M2, measured for *w*_wg_ = 400 nm. The Lorentzian fit of the cavity mode resonance is displayed in dashed line for reference. Power spectral density at mechanical resonance *S*_P_(*Ω*_m_, Δ/*κ*) (open circles) as a function of the normalised detuning Δ/*κ* for modes (**c**) M1 and (**d**) M2 and for the same *w*_wg_. The solid lines represent the fit to the theoretical model. Contributions to transmission spectrum noise plotted in arbitrary units (blue: dispersive coupling; orange: intrinsic dissipative coupling; yellow: external dissipative coupling) for (**e**) M1 and (**f**) M2 versus Δ/*κ*. The inferred optomechanical coupling rates are indicated.

To gain an insight into the dependence of such coupling mechanisms on the geometric features of the access waveguides, we performed a detailed analysis of the evolution of *S*_P_(*Ω*_m_, Δ/*κ*), measured as a function of the normalised laser detuning Δ/*κ*, on all the waveguide widths *w*_wg_ present on our sample (all the other geometric parameters of the optomechanical resonators were kept constant). In the case of a purely dispersive coupling, *S*_P_(*Ω*_m_, Δ/*κ*) should be proportional to (∂*T*/∂Δ)^2^, yielding a symmetric curve with respect to Δ/*κ* = 0 with a null mechanical amplitude at the optical resonance wavelength. Conversely, in the case of a purely dissipative coupling, the optomechanical amplitude is expected to exhibit its maximum around zero detuning. In Fig. 2(a,b), we show *S*_P_(*Ω*, Δ/*κ*) for the two lowest-frequency mechanical modes, which we refer to as M1 and M2 [see Fig. 1(d)], for *w*_wg_ = 400 nm. For both modes, the mechanical frequency shifts as a result of the optical spring effect. However, unlike systems in which the optomechanical interaction is generated by intracavity fields, for which the optical spring effect vanishes at zero detuning^7^, our device shows the largest mechanical frequency shift around the PhC resonance, suggesting a significant dissipative coupling^38^. This is confirmed by the strong asymmetry of *S*_P_(*Ω*_m_, Δ/*κ*) with respect to a zero-laser detuning – evaluated at the mechanical resonances *Ω*_m_ – for M1 and M2^49^, as illustrated in Fig. 2(c,d), respectively. By fitting the experimental data to Eq. (1), we could extract the optomechanical coupling coefficients for M1 and M2 (see Methods for more details on the fitting procedure).

The optomechanical coupling coefficients and the respective relative contributions to the detected signals (*g*_*ω*_∂*T*/∂Δ in blue, *g*_*κ*,e_∂*T*/∂*κ*_e_ in yellow and *g*_*κ*,i_∂*T*/∂*κ*_i_ in orange) are shown in Fig. 2(e,f). For both modes, the coupling coefficients deduced from the fits confirm the importance of the dissipative coupling contribution, whose relative weight is peculiar to each mode, as suggested by the different relative contributions to the detected optomechanical response, shown in Fig. 2(e,f).

**Figure 3:**
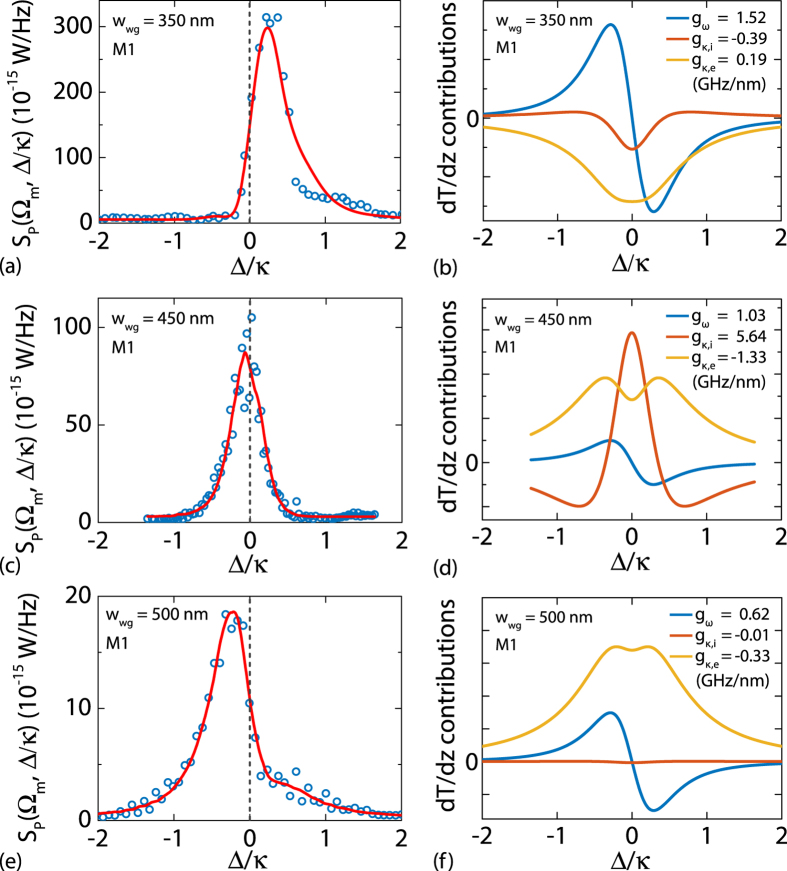
Optomechanical response for a varying *w*_wg_. (**a,c,e**) Power spectral densities at mechanical resonance (circles: experimental data; solid lines: fit to model) *S*_P_(*Ω*_m_, Δ/*κ*) and (**b,d,f**) relative contributions of coupling dispersive (blue curves) and dissipative (orange curves: intrinsic; yellow curves: external) coupling mechanisms (arbitrary units). (**a,b**) *w*_wg_ = 350 nm. (**c,d**) *w*_wg_ = 450 nm. (**e,f**) *w*_wg_ = 500 nm.

The flexibility of the sample design permitted by our integrated approach enables us to investigate in detail the variation of the optomechanical coupling mechanisms as a function of the waveguide width *w*_wg_, while keeping the membrane suspension height *h* constant. In Fig. 3(a,c,e) we illustrate the dependence of the transduction of optomechanical resonators on the waveguide width, for *w*_wg_ = 350 nm, 450 nm, and 500 nm, respectively. In all cases, the strong non-zero optomechanical signal at the optical resonance confirms the importance of the dissipative coupling mechanism for our optomechanical resonators. However, the relative weight of the different coupling mechanisms varies as a function of *w*_wg_. In particular, Fig. 3(b,d,f) reveal a comparatively higher dispersive component for both *w*_wg_ = 350 nm and *w*_wg_ = 500 nm. Conversely, *w*_wg_ = 450 nm is characterized by the highest dissipative contribution to the detected signal, as attested by the mechanical amplitude peaking at zero laser detuning. Our fits also suggest a certain variability of *κ*_i_ as a function of *w*_wg_, which we attribute to defects introduced during the epitaxial growth or during the fabrication process of the optomechanical resonators.

**Figure 4:**
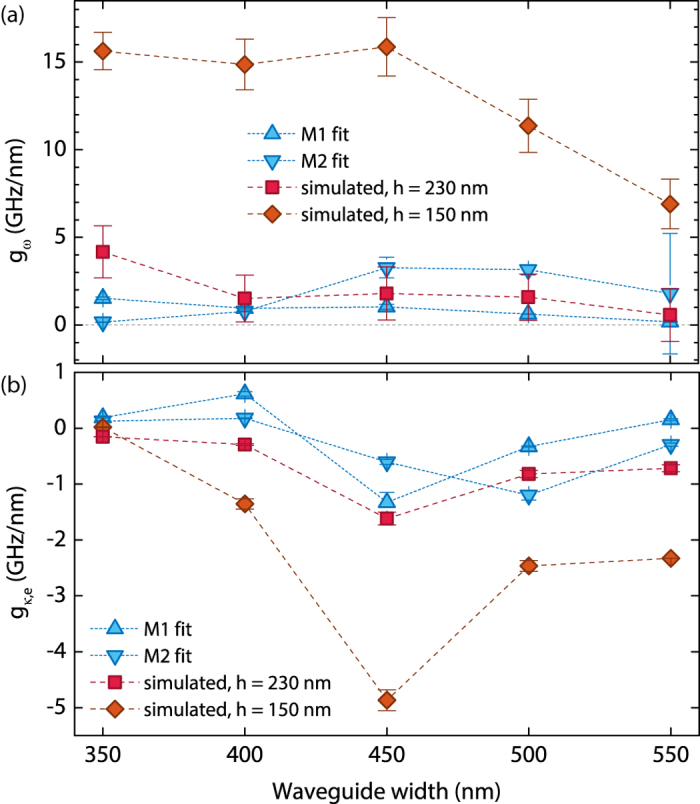
Comparison between simulated and experimental coupling coefficients. (**a**) Dispersive coupling coefficient *g*_*ω*_ and (**b**) external dissipative coupling coefficient *g*_*κ*,e_ plotted against waveguide width *w*_wg_. Blue, up-pointing triangles: fit of experimental points, M1. Blue, down-pointing triangles: fit of experimental points, M2. Red squares: simulated values, air gap of 230 nm. Orange diamonds: computed values, air gap of 150 nm.

The values of *g*_*ω*_ and of *g*_*κ*,e_ extracted from fitting the experimental data to Eq. (1) as a function of *w*_wg_ both for M1 and for M2 are summarised in Fig. 4(a,b), respectively. The observed trend of these two quantities is directly related to the shape and the width of the optomechanical response *S*_P_(*Ω*_m_, Δ/*κ*).

### Simulation of optomechanical coupling

A frequently used approach to achieve a deeper understanding of the physical mechanisms governing the optomechanical coupling consists in quantifying the shift of the PhC cavity mode resonance and of the coupling rate between the PhC cavity and the waveguide by perturbation theory. In a perturbative approach, the PhC cavity resonance shift induced by a close-by waveguide is evaluated by computing the overlap integral of the unperturbed PhC cavity mode pattern with the variation to the unperturbed dielectric constant distribution induced by the presence of the waveguide. The cavity loss rate induced by the presence of the waveguide can be obtained by computing the overlap between the unperturbed PhC cavity mode pattern and the unperturbed waveguide mode pattern^52^. A more thorough (albeit more computationally demanding) approach relies on performing static 3D FDTD computations of the full system comprising both the PhC cavity and the waveguide. We ran 3D FDTD simulations for all the five values of *w*_wg_ present on our sample, with the scope of predicting the dispersive and the external dissipative coupling coefficients of our optomechanical resonators. The distance between the PhC cavity and the waveguide was defined by the air gap *h* between the lower edge of the InP membrane and the upper edge of the Si ridge. The computations were performed with an adaptative mesh refinement, allowing for a variation of *h* in steps of 10 nm, to obtain the dependence of the cavity resonance wavelength *λ*_c_ and of the PhC cavity loss rate *κ* as a function of *h*. The derivatives of these curves were subsequently evaluated at a suspension height *h* = 230 nm, corresponding to the measured separation between the InP membrane and the waveguides on the fabricated sample, providing the computed *g*_*ω*_ and *g*_*κ*,e_. In the case of the computed cavity linewidth, to account for deviations from the ideal simulated structures, related to the presence of fabrication-induced defects in the optomechanical resonators, the *κ* versus *h* curves have been renormalised (keeping the same field decay length of the PhC cavity mode in the out-of-plane direction) such that they pass through the experimentally determined *κ* at *h* = 230 nm. The computed *g*_*ω*_ shows the same trend as the experimentally determined *g*_*ω*_ for M1, whose values fall within the error bars of the computed coupling coefficients, except for *w*_wg_ = 350 nm. A good agreement between the simulated *g*_*ω*_ and the experimental *g*_*ω*_ is also observed for M2, although M1 is the mechanical mode whose displacement pattern mainly resembles the simulated translation of the InP membrane in the out-of-plane direction. The non-monotonic trend observed for the computed *g*_*κ*,e_ versus *w*_wg_, with 3D FDTD simulations, results from a better phase-matching condition achieved between the L_3_ PhC cavity (with a renormalized *κ* as explained above to take into account the experimentally observed optical loss rate) and a waveguide with *w*_wg_ = 450 nm. This trend is also very similar to the corresponding experimentally determined coupling coefficient for M1, as shown in Fig. 4(b).

A further advantage of our heterogeneous integration approach is the possibility to vary *h* by properly adjusting the thickness of the SiO_2_ layer deposited on top of the InP substrate, to achieve a predefined optomechanical coupling. The computed *g*_*ω*_ and *g*_*κ*,e_ for *h* = 150 nm are compared for reference with the computed coupling coefficients for *h* = 230 nm in Fig. 4(a,b), respectively. Significantly larger dispersive and dissipative optomechanical coupling coefficients may be therefore expected in such optomechanical resonators, at the expenses of a broader PhC cavity mode.

## Discussion

In the previous sections, we have demonstrated InP optomechanical resonators, consisting of a L_3_ PhC nanocavity etched in a thin InP membrane, heterogeneously integrated onto a SOI waveguide substrate, exhibiting tailored dispersive and dissipative optomechanical coupling. Flexural mechanical modes in the MHz range were systematically observed on the investigated devices. By performing systematic measurements of the optomechanical response of the fabricated optomechanical resonators as a function of the probe laser wavelength, we showed consistently that the most significant contribution to the optomechanical transduction was of dissipative nature. We observed a different relative sign of the dispersive and external dissipative coupling coefficients by varying the waveguide width (see Fig. 4), which can be regarded as a way to finely tailor the optomechanical coupling strength. Simulations also demonstrate that our system has an additional ‘knob’ to coarsely tune the optomechanical coupling, represented by the suspension height of the membrane.

In the case of a non-zero dissipative coupling, cooling even in the unresolved-sideband regime is permitted^44^. The tailored optomechanical coupling exhibited by our optomechanical system makes it potentially capable of reaching the optimal mixed coupling (i.e. dispersive-to-dissipative coupling ratio, including the correct relative sign) yielding the minimal phonon number^44^, paving the way to an optimal mechanical mode cooling in resonators featuring simultaneously dispersive and dissipative coupling and operating in the unresolved-sideband regime.

This fabrication approach is intrinsically up-scalable, leading to potential applications in optomechanical circuits at the full chip level. In perspective, a first multi-element component, which could be fabricated by following our approach, may include multiple InP membranes, having different resonance wavelengths, aligned on top of a single waveguide, potentially allowing for a simultaneous multiple-device addressing through the same channel. The hybrid integration approach demonstrated here could enable us to bring together the strengths of quantum light emitters embedded in a III-V semiconductor matrix and of optomechanical signal processors for the realization of hybrid optomechanical devices.

## Methods

### Sample fabrication

The SOI waveguide substrate, on which the access waveguides used to couple light into and out of the PhC cavities were fabricated, is composed of a 2 μm thick buried SiO_2_ layer, capped by a 220 nm thick Si waveguiding layer. This substrate is etched to a depth of 220 nm, leaving Si ridges of width *w*_wg_ ranging from 350 nm to 550 nm, yielding single mode waveguides for TE polarization around the 1550 nm band. Grating couplers, fabricated at each end of the 6 mm-long waveguides, allow for vertical coupling of light into and out of the chip^53^. The 260 nm thick InP membranes (embedding a layer of self-assembled InAsP quantum dots in the middle for PhC cavity characterization^18^) are grown, along with a 1 μm thick InGaAs etch-stop layer, on top of an InP (100) substrate by MetalOrganic Vapour Phase Epitaxy. The III-V and the SOI substrates are bonded by depositing a 200 nm thick layer of SiO_2_ on the InP membrane and by spin-coating the SOI chip with a 250 nm thick layer of DiVinylSiloxane-BenzoCycloButene (DVS-BCB), thereby planarizing the SOI substrate and leaving a residual 30 nm thick layer of DVS-BCB on top. Bonding is achieved by pressing the two substrates together and by curing the DVS-BCB at 320 °C. Chemical etching of the InP substrate and of the InGaAs etch-stop layer leaves the 260 nm thick InP layer bonded on top of the SOI substrate. The membrane is subsequently patterned by a standard combination of electron-beam lithography (EBL), reactive ion etching and inductively coupled plasma etching, using a SiN layer as rigid mask. The alignment of the PhC cavities with respect to the SOI level is performed by making use of alignment markers etched beforehand in the SOI substrate. An alignment accuracy between the SOI waveguides and the PhC cavities of ≲40 nm is routinely achieved^54^. The excess InP membrane is then etched, by employing a layer of EBL resist to protect the PhC cavities and the supporting structures. This results in InP mesas holding the PhC optomechanical resonators [see SEM image in Fig. 1(b)]. The access waveguide is visible in the SEM image as a bright straight line running beneath the suspended InP membrane. The InP membrane is fully suspended by wet chemical etching of the underlying SiO_2_ layer, followed by a critical point drying step. The lateral InP suspension pads act as protective structures for the SiO_2_ layer beneath them, leaving them anchored to the substrate.

### Photonic crystal design

L_3_ cavities, obtained by omitting three PhC holes along a line of an otherwise perfect hexagonal lattice of constant *a* = 420 nm, were fabricated on the InP membrane. The hole radius *r* was designed using a commercial software implementing the 3D Finite Difference in Time Domain (FDTD) method, such that the cavity mode was at approximately 1560 nm, resulting in *r* ~ 100 nm. The holes at the end of the cavity were shifted outwards by *d* = 0.2*a* to optimize the optical quality factor *Q*^45^. The cavity mode was characterized by performing micro-photoluminescence spectroscopy in the absence of the access waveguides, yielding an unloaded *Q* ~ 10^4^.

### Optomechanical resonator characterization

The measurement setup is sketched in Fig. 1(c). The light emitted from a tunable laser diode is used for the readout of the mechanical modes of the PhC membrane. The polarization of the light source is adjusted before coupling it into the access waveguide via the input grating coupler. The light passing through the cavity is outcoupled via the output grating coupler situated at the opposite end of the waveguide. This signal could be directed either to a spectrometer (for transmission spectrum measurements) or, after proper filtering and amplification, to a fast photodiode (3.5 GHz bandwidth) connected to an electrical spectrum analyzer, to measure the mechanical spectra of the InP membranes. All the measurements were performed at room temperature. The sample was kept in a vacuum chamber, at a pressure of <1.0 × 10^−4^ mbar, to avoid air damping effects.

### Fitting procedure

In Eq. (1), 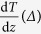 requires a total of five free parameters. Among them, *κ* and *κ*_i_ are purely optical parameters; these parameters are extracted from optical micro-photoluminescence and transmission measurements, respectively on samples without and with waveguide circuitry. Dispersive (*g*_*ω*_) and dissipative (*g*_*κ*,e_ and *g*_*κ*,i_) coupling rates are obtained by fitting the power spectral density at mechanical resonance *S*_P_(*Ω*_m_, Δ/*κ*) to Eq. (1). Asymmetric evolution of *S*_P_(*Ω*_m_, Δ/*κ*) with respect to zero laser detuning (Δ/*κ* = 0) suggests a significant dispersive coupling contribution to the optomechanical response, while symmetric behavior suggests a dominant dissipative coupling. We thus applied a two-step fitting procedure by first cancelling the weaker coupling rate and get a rough estimate of the dominant ones, and, in a second step, by letting all three parameters free in order to improve the reliability of the fit.

## Additional Information

**How to cite this article**: Tsvirkun, V. *et al.* Integrated III-V Photonic Crystal – Si waveguide platform with tailored optomechanical coupling. *Sci. Rep.* **5**, 16526; doi: 10.1038/srep16526 (2015).
